# Characteristics of Persons Who Report Using Only Nicotine-Containing Products Among Interviewed Patients with E-cigarette, or Vaping, Product Use–Associated Lung Injury — Illinois, August–December 2019

**DOI:** 10.15585/mmwr.mm6903e1

**Published:** 2020-01-24

**Authors:** Isaac Ghinai, Livia Navon, Jayleen K.L. Gunn, Lindsey M. Duca, Sarah Brister, Sarah Love, Rachel Brink, Geroncio Fajardo, Jona Johnson, Lori Saathoff-Huber, Brian A. King, Christopher M. Jones, Vikram P. Krishnasamy, Jennifer E. Layden

**Affiliations:** ^1^Illinois Department of Public Health; ^2^Epidemic Intelligence Service, CDC; ^3^Center for Preparedness and Response, CDC; ^4^National Center for HIV/AIDS, Viral Hepatitis, STD and TB Prevention, CDC; ^5^National Center for Chronic Disease Prevention and Health Promotion, CDC; ^6^Council of State and Territorial Epidemiologists; ^7^National Center for Emerging and Zoonotic Infectious Diseases, CDC; ^8^Agency for Toxic Substances and Disease Registry, CDC; ^9^National Center for Injury Prevention and Control, CDC.

In 2019, the United States experienced an outbreak of e-cigarette, or vaping, product use–associated lung injury (EVALI) (*1*). Most EVALI patients have reported using tetrahydrocannabinol (THC)-containing e-cigarette, or vaping, products obtained from informal sources (*2*,*3*), and vitamin E acetate in these products has been closely linked with EVALI (*4*,*5*). However, some EVALI patients report using only nicotine-containing products. This study compared demographic, product use, and clinical characteristics of EVALI patients in Illinois who reported using only nicotine-containing e-cigarette, or vaping, products with those of patients who reported using any THC-containing products. Among 121 interviewed Illinois EVALI patients, 17 (14%) reported using only nicotine-containing products, including nine (7%) patients who had no indication of any THC use, based on self-report or toxicology testing. Compared with patients who used any THC-containing products, these nine patients were significantly more likely to be older and female and were less likely to experience constitutional symptoms or to have leukocytosis on initial evaluation. Although vitamin E acetate has been strongly linked with EVALI, evidence is not sufficient to rule out the contribution of other chemicals of concern, including chemicals in either THC- or non-THC-containing products, in some reported EVALI cases. The contributing cause or causes of EVALI for patients reporting use of only nicotine-containing products warrants further investigation.

Medical records were requested for all suspected EVALI cases reported to the Illinois Department of Public Health (IDPH), and clinical information was abstracted using a standardized form. Cases were included in this study if the illness met the EVALI surveillance definition for a confirmed or probable case.* EVALI patients or their proxies were also asked to complete a structured questionnaire that collected information about demographics and e-cigarette, or vaping, product use. A follow-up interview was attempted with all patients who reported that they did not use THC-containing products on the initial questionnaire to confirm that they used only nicotine-containing products, and corresponding medical records were reexamined for any indication of THC use (e.g., a positive urine cannabinoid screen or report of smoking combustible marijuana to a health care provider). When available, bronchoalveolar lavage fluid specimens were sent to CDC for laboratory testing. EVALI cases reported during July 20–December 1, 2019, with a completed initial structured questionnaire were included in this analysis.

Among 195 EVALI cases reported to IDPH, 121 patients (62%) had a completed structured questionnaire. These patients were categorized into two analysis groups: those who reported using any THC-containing products and those who reported using no THC-containing products and reported using only nicotine-containing products. The group that reported no THC-containing product use was further stratified into two groups: those with no indication of any THC use after follow-up interview and reexamination of medical records and those who reported no THC-containing e-cigarette, or vaping, product use but who did have evidence of using THC (e.g., disclosed use of combustible marijuana or had a positive urine cannabinoid screen).

Demographic characteristics, use of nicotine-containing products, and clinical characteristics of patients with no indication of any THC use were compared with those who reported any THC-containing product use. To allow replication analyses by other health departments, where access to the follow-up interviews or medical records necessary to define the subgroups compared here might not be available, patients who reported using no THC-containing products (whether or not they had subsequent indication of any THC use) were also compared with those patients who reported using THC-containing products. Differences were assessed using Pearson’s chi-squared test or Fisher’s exact test for cells with <5 observations. The Wilcoxon rank sum test was used to compare medians. P-values <0.05 were considered statistically significant. Analyses were conducted using SAS (version 9.4; SAS Institute).

Initially, 19 (16%) of 121 interviewed Illinois EVALI patients reported using only nicotine-containing products (Figure), nine of whom participated in a follow-up interview; at that time, two patients (both aged <18 years) disclosed that they used products likely to have contained THC. Both patients completed the initial questionnaire with parents present, whereas follow-up interviews were conducted privately (with parental consent). Thus, overall, 104 patients (86%) reported using any THC-containing products, and 17 (14%) reported using only nicotine-containing products. Six of the 17 patients who reported using only nicotine-containing products also reported smoking combustible marijuana, and two other patients had positive urine cannabinoid screens and reported combustible marijuana use to their health care providers; bronchoalveolar lavage fluid from one of these patients was available for testing, and both THC and vitamin E acetate were detected in the fluid. Thus, nine of 121 patients (7%) had no indication of any THC use and constituted the analysis subgroup; three of these patients underwent urine cannabinoid screening and all were negative.

**FIGURE:**
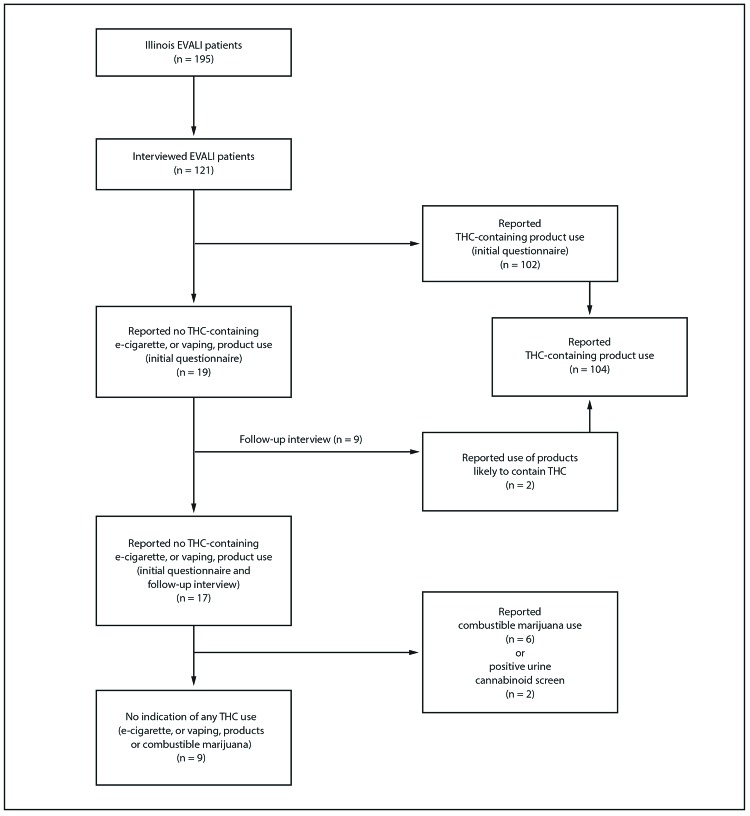
Categorization of patients with confirmed and probable e-cigarette, or vaping, product use–associated lung injury (EVALI), by tetrahydrocannabinol (THC)-containing product use — Illinois, July–December 2019

Among 104 patients who reported using any THC-containing products, 46 (44%) were classified as having confirmed EVALI, compared with two of 17 patients (12%) who reported using only nicotine-containing products, and none of nine patients with no indication of any THC use (p = 0.01 for both) (Table 1). For the most part, these nine patients did not meet the minimum criteria for negative infectious disease testing to be classified as a confirmed case, in some instances because infection was not considered in the differential diagnosis. Compared with patients who reported using any THC-containing products, patients with no indication of any THC use were more likely to be female (78% versus 25%; p<0.01) and aged ≥45 years (33% versus 2%, p<0.01). There were no statistically significant differences in the frequency of use of nicotine-containing products, number of nicotine-containing products used, or source of the nicotine-containing products.

**TABLE 1:** Demographic characteristics and use of nicotine-containing e-cigarette, or vaping, products among patients with e-cigarette, or vaping, product use–associated lung injury (EVALI), by tetrahydrocannabinol (THC)-containing product use — Illinois, July–December 2019

Characteristic	No. (%)		p-value^¶^	No. (%)	p-value^¶^
	Reported THC-containing product use* (reference)	Reported no THC-containing product use^†^		No indication of any THC use^§^	
Total (N = 121)**	**104 (86)**	**17 (14)**	**—**	**9 (7)**	—
**Case status**					
Confirmed	46/104 (44)	2/17 (12)	0.01	0/9 (0)	0.01
Probable	58/104 (56)	15/17 (88)		9/9 (100)	
**Gender**					
Female	26/104 (25)	7/17 (41)	0.2	7/9 (78)	0.003
**Age group (yrs)**					
13–24	65/104 (63)	7/17 (41)	0.007	2/9 (22)	<0.001
25–44	37/104 (36)	7/17 (41)		4/9 (44)	
45–74	2/104 (2)	3/17 (18)		3/9 (33)	
**Race/Ethnicity**					
White, non-Hispanic	61/93 (66)	11/16 (69)	0.3	4/9 (44)	0.06
Black, non-Hispanic	6/93 (6)	3/16 (19)		3/9 (33)	
Other, non-Hispanic	9/93 (10)	1/16 (6)		1/9 (11)	
Hispanic	17/93 (18)	1/16 (6)		1/9 (11)	
**Nicotine-containing e-cigarette, or vaping, products**					
Used nicotine product >5 times per day	35/48^††^ (73)	8/15 (53)	0.2	4/8 (50)	0.2
Used more than one nicotine product	13/55 (24)	3/17 (18)	0.7	2/9 (22)	1.0
**Source of nicotine product (s)^§§^**					
Vape or tobacco shop	22/47 (47)	10/14 (71)	0.1	7/9 (78)	0.1
Convenience store	14/47 (30)	5/14 (36)	0.7	2/9 (22)	1.0
Online	4/47 (9)	1/14 (7)	1.0	1/9 (11)	1.0
Informal source^¶¶^	11/47 (23)	0/14 (0)	0.06	0/9 (0)	0.2

* Patients who reported using THC-containing e-cigarette, or vaping, products on initial structured questionnaire or follow-up interview.

^†^ Patients who reported not using THC-containing e-cigarette, or vaping, products on initial structured questionnaire and follow-up interview.

^§^ Subgroup of patients who reported not using THC-containing products who also had no indication of use of any other THC-containing substance (e.g., reported not smoking combustible marijuana, had negative toxicology testing, if performed).

^¶^ P-values for comparisons, using Pearson’s chi-squared test or Fisher’s exact test (for cells with <5 observations). Statistical tests compared EVALI patients who reported THC-containing product use with EVALI patients who reported no THC-containing product use and with EVALI patients with no indication of any THC use.

** Data were not available for all variables for all patients. Differing denominators reflect missing data.

^††^ Only patients who used nicotine-containing e-cigarette, or vaping, products and reported a frequency of use are included in the denominator.

^§§^ Six patients reported purchasing nicotine-containing e-cigarette, or vaping, products from more than one source: vape/tobacco shop and convenience store (three) and vape/tobacco shop and online (three).

^¶¶^ Informal sources of nicotine-containing products include friends, family members, or from in-person or online dealers.

At initial hospital evaluation, patients with no indication of any THC use were less likely than were patients who reported using any THC-containing products to experience constitutional symptoms (56% versus 96%; p<0.01), have an initial leukocytosis (38% versus 91%; p<0.01), or to have presented to an outpatient provider or emergency department before hospitalization (25% versus 80%; p<0.05) (Table 2). There were no statistically significant differences between patients with no indication of any THC use and those who reported using any THC-containing product in initial vital signs, other initial laboratory results, admission to an intensive care unit, or severe outcome (defined as death or respiratory failure requiring endotracheal intubation and mechanical ventilation).

**TABLE 2:** Clinical characteristics of patients with e-cigarette, or vaping, product use-associated lung injury (EVALI), by reported tetrahydrocannabinol (THC)-containing product use — Illinois, July–December 2019

Characteristic	No. (%)		p-value^¶^	No. (%)	p-value^¶^
	Reported THC-containing product use* (reference)	Reported no THC-containing product use^†^		No indication of any THC use^§^	
Total (N = 121)**	**104 (86)**	**17 (14)**	**—**	**9 (7)**	**—**
**Past medical history**					
Existing respiratory condition^††^	12/61 (20)	3/14 (21)	1.0	2/7 (29)	0.6
Existing cardiovascular condition^§§^	2/61 (3)	1/14 (7)	0.5	1/7 (14)	0.3
**Symptoms reported at presentation**					
Any respiratory symptom^¶¶^	99/100 (99)	16/17 (94)	0.3	8/9 (89)	0.2
Any gastrointestinal symptom***	88/100 (88)	14/17 (82)	0.5	7/9 (78)	0.3
Any constitutional symptom^†††^	96/100 (96)	13/17 (76)	0.02	5/9 (56)	0.001
**Vital signs at presentation**					
Hypoxemia (O_2_ saturation ≤95% on room air)	66/104 (63)	10/17 (59)	0.7	5/9 (56)	0.7
Tachypnea (RR >20 breaths per minute)	25/66 (38)	7/15 (47)	0.5	3/8 (38)	1.0
Tachycardia (HR >100 beats per minute)	40/68 (59)	7/15 (47)	0.7	4/8 (50)	0.7
Fever (temperature ≥100.4°F [38°C])	21/65 (32)	3/14 (21)	0.5	2/8 (25)	1.0
**Initial laboratory results**					
Leukocytosis (WBC count >11,000 per mm^3^)	63/69 (91)	9/16 (56)	0.001	3/8 (38)	0.001
with >80% neutrophils	53/63 (84)	5/9 (56)	0.07	1/3 (33)	0.08
Sodium <135 mmol/liter	17/69 (25)	3/16 (19)	0.8	0/8 (0)	0.2
Potassium <3.5 mmol/liter	18/68 (26)	2/15 (13)	0.5	2/7 (29)	1.0
AST or ALT >35 U/liter	27/61 (44)	9/13 (69)	0.1	5/6 (83)	0.1
**Clinical course**					
Duration of symptoms before hospitalization (median days, range)	7 (1–148)	4 (0–205)	0.04	3 (0–205)	0.1
Outpatient or ED visit before hospitalization	51/64 (80)	3/10 (30)	0.003	1/4 (25)	0.04
Received glucocorticoids	53/55 (96)	8/10 (80)	0.1	5/5 (100)	1.0
Clinical improvement documented after glucocorticoids	16/53 (30)	1/8 (13)	0.4	0/5 (0)	0.3
Admitted to intensive care unit	40/81 (49)	9/17 (53)	0.8	5/9 (56)	1.0
Severe outcome^§§§^	19/90 (21)	7/17 (41)	0.07	4/9 (44)	0.2

**Abbreviations:** ALT = alanine aminotransferase; AST = aspartate aminotransferase; ED = emergency department; HR = heart rate; O_2_ = oxygen; RR = respiratory rate; WBC = white blood cell.

* Patients who reported using THC-containing e-cigarette, or vaping, products on initial structured questionnaire or follow-up interview.

^†^ Patients who reported not using THC-containing e-cigarette, or vaping, products on initial structured questionnaire or follow-up interview.

^§^ Subgroup of those patients who reported not using THC-containing products who also had no indication of use of any other THC-containing substance (e.g., reported not smoking combustible marijuana, had negative toxicology testing, if performed).

^¶^ P-values for comparisons, using Pearson’s chi-squared test or Fisher’s exact test (for cells with <5 observations). Wilcoxon rank sum test used to compare medians. Statistical tests compared EVALI patients who reported THC-containing product use with EVALI patients who reported no THC-containing product use and with EVALI patients with no indication of any THC use.

** Data were not available for all patients. Differing denominators reflect missing data.

^††^ Existing respiratory conditions include asthma, chronic obstructive pulmonary disease, bronchitis, previous lung cancer and obstructive sleep apnea.

^§§^ Existing cardiovascular conditions include ischemic heart disease, congestive heart failure and congenital heart disease.

^¶¶^ Respiratory symptoms include shortness of breath, any cough, pleuritic chest pain.

*** Gastrointestinal symptoms include nausea, vomiting, diarrhea, abdominal pain.

^†††^ Constitutional symptoms include subjective fever, chills, weight loss, fatigue/malaise.

^§§§^ Severe outcomes include death or respiratory failure requiring endotracheal intubation and mechanical ventilation.

## Discussion

Among the 121 EVALI patients included in this analysis, nine (7%) reported using only nicotine-containing e-cigarette, or vaping, products and had no indication of any THC use. EVALI patients who had no indication of any THC use were more likely to be older and female and less likely to have constitutional symptoms and an initial leukocytosis and to have seen an outpatient provider before hospitalization. Vitamin E acetate has been strongly linked to the EVALI outbreak (*4*); however, before the current EVALI outbreak, there have been case reports of lung injury associated with nicotine-containing e-cigarette, or vaping, product use (*6*,*7*). Along with a longstanding baseline rate of emergency department visits from e-cigarette, or vaping, product use identified from syndromic surveillance (*8*), these findings suggest that some EVALI cases might be associated with the use of nicotine-containing products. Given the different demographics, clinical presentations, and the lack of any indication of exposure to THC-containing products, the contributing cause or causes of EVALI for persons using only nicotine-containing products might differ from the majority of EVALI patients and warrants further investigation.

A small number of EVALI patients in Illinois who initially reported not using THC-containing e-cigarette, or vaping, products were ultimately determined to have used these products through follow-up interview and laboratory testing. These findings demonstrate inconsistencies in patient reporting of THC-containing e-cigarette, or vaping, product use. Empathetic and private questioning might help facilitate accurate reporting, particularly among younger patients (*9*). In all suspected EVALI patients, providers should consider conducting, with informed consent, urine toxicology testing, including testing for THC *(**10**)*.

The findings in this report are subject to at least four limitations. First, findings should be interpreted with caution because of the small number of patients who reported not using any THC-containing products. This small sample size limits the statistical power or ability to account for potential confounding factors. Second, product use was self-reported and might be subject to reporting biases, particularly given that recreational use of THC-containing products was illegal in Illinois before January 1, 2020. Moreover, urine toxicology screens and laboratory testing of e-cigarette, or vaping, products were not performed routinely. Thus, the group of 17 patients who reported not using THC-containing products includes both persons with and without exposure to THC; nevertheless, this group was included in this report principally to aid future analyses. The primary comparison in the analysis reported here is between those who report using THC-containing products and those with no indication of any THC use. Nevertheless, of the nine patients analyzed in this report as having no indication of any THC use, only three were screened for cannabinoids. Third, because EVALI has diverse presentations and an intentionally sensitive surveillance case definition, some non-cases might have been misclassified as cases. Finally, not all EVALI patients were reached for initial or follow-up interview, which could limit generalizability of these findings.

CDC recommends that persons should not use THC-containing e-cigarette, or vaping, products, particularly those obtained from informal sources such as friends, family members, or from in-person or online dealers. Vitamin E acetate is strongly linked to the EVALI outbreak. However, evidence is not sufficient to rule out the contribution of other chemicals of concern, including chemicals in either THC- or non-THC-containing products, in some reported EVALI cases. Vitamin E acetate should not be added to e-cigarette, or vaping, products. In addition, persons should not add any other substances not intended by the manufacturer to e-cigarette, or vaping, products, including products purchased through retail establishments.^†^ Adults using nicotine-containing e-cigarette or vaping products as an alternative to cigarettes should not go back to smoking; they should weigh all available information and consider using Food and Drug Administration–approved cessation medications.^§^ They should contact their health care provider if they need help quitting tobacco products, including e-cigarettes, as well as if they have concerns about EVALI. Adults who do not currently use tobacco products should not start using e-cigarette, or vaping, products. Finally, e-cigarette, or vaping, products should never be used by youths, young adults, or pregnant women.

**Summary**

**What is already known about this topic?**

Most patients with e-cigarette, or vaping, product use–associated lung injury (EVALI) report using tetrahydrocannabinol (THC)-containing products. However, some report using only nicotine-containing products.

**What is added by this report?**

Among 121 interviewed Illinois EVALI patients, nine who reported using only nicotine-containing products and had no indication of any THC use were more likely to be older, female, and less likely to experience constitutional symptoms or leukocytosis than were patients who used THC-containing products.

**What are the implications for public health practice?**

Although vitamin E acetate has been strongly linked with EVALI, evidence is not sufficient to rule out the contribution of other chemicals of concern, including chemicals in either THC- or non-THC-containing products, in some reported EVALI cases.

## Footnotes

* https://www.cdc.gov/tobacco/basic_information/e-cigarettes/severe-lung-disease/health-departments/index.html#primary-case-def.

† https://www.cdc.gov/tobacco/basic_information/e-cigarettes/severe-lung-disease/need-to-know/index.html#cdc-recommends.

§ https://www.cdc.gov/tobacco/campaign/tips/quit-smoking/index.html?s_cid.
